# Cassava Trait Preferences of Men and Women Farmers in Nigeria: Implications for Breeding

**DOI:** 10.1007/s12231-018-9421-7

**Published:** 2018-07-12

**Authors:** Béla Teeken, Olamide Olaosebikan, Joyce Haleegoah, Elizabeth Oladejo, Tessy Madu, Abolore Bello, Elizabeth Parkes, Chiedozie Egesi, Peter Kulakow, Holger Kirscht, Hale Ann Tufan

**Affiliations:** 1 International Institute of Tropical Agriculture (IITA), Ibadan, Nigeria; 2 Council on Science and Industrial Research-Crops Research Institute (CSIR-CRI), Accra, Ghana; 3 National Root Crops Research Institute (NRCRI), Umudike, Nigeria; 4 Deutsche Gesellschaft für Internationale Zusammenarbeit (GIZ), Bonn, Germany; 5 Cornell University, Ithaca, New York USA

**Keywords:** plant breeding, cassava, gender, trait preferences, Nigeria

## Abstract

Nigeria is the world’s largest cassava producer, hosting a diverse array of cassava farmers and processors. Cassava breeding programs prioritize “common denominator” traits in setting breeding agendas, to impact the largest possible number of people through improved varieties. This approach has been successful, but cassava adoption rates are less than expected, with room for improvement by integrating traits in demand by farmers and processors. This paper aims to inform breeding priority setting, by examining trait and varietal preferences of men and women cassava farmer/processors. Men and women in eight communities in Southwest and Southeast Nigeria were consulted using mixed methods. Women and men had significantly different patterns of cassava use in the Southwest. Fifty-five variety names were recorded from the communities demonstrating high genetic diversity maintained by growers, especially in the Southeast. High yield, early maturity, and root size were most important traits across both regions, while traits women and men preferred followed gender roles: women prioritized product quality/cooking traits, while men placed higher priority on agronomic traits. Trait preference patterns differed significantly between the Southeast and Southwest, and showed differentiation based on gender. Patterns of access to stem sources were determined more by region and religion than gender.

## Introduction

Cassava **(***Manihot esculenta* Crantz) is a major staple crop in Nigeria, engaging over four million farmers in production and providing food for over 100 million persons (FAOSTAT 2016). Cassava storage roots provide an efficient source of carbohydrate food energy, cultivated widely for its ability to withstand harsh environmental and agronomic conditions as well as to its utilization as raw material for many uses and food products (Akoroda 1995). Major cassava based food products consumed in Nigeria are *gari, fufu* and *lafun*. Most of these cassava food products are made by farmers themselves, who process and consume the crop (IITA 2012). This joint small-scale farming and processing represents by far the largest cassava food product production in Nigeria (Forsythe et al. 2016; Onyenwoke and Simonyan 2014).

Studies of farmers’ preferences for crop variety traits of food crops, many of which are economically important, show that such preferences are not uniform but vary in relation to the agronomic, technical, and socio-cultural context of the modes of production and processing (Smale et al. 2001). Differential trait preferences also follow gender divisions of labor and market access, observed across multiple crops in sub-Saharan Africa (Christinck et al. 2017). In most parts of rural Nigeria, division of labor for agricultural production is gender-specific and varies by age (Mohammed and Abdulquadri 2012). However, these roles vary by ethnic groups and regions (FAO 2011). Women play a central role in Nigerian cassava production, processing and marketing (Enete et al. 2002), and provide much of the labor associated with cassava production. In particular women perform the majority of cassava processing in Nigeria (Curran et al. 2009; Walker et al. 2014). As such, cassava is often defined as a “woman’s crop” (Forsythe et al. 2015). For example, as commercialization of cassava increases, men’s participation in production and processing increases (Nweke et al. 2002), necessitating greater gender analysis of cassava production, processing and in particular commercialization to ensure new interventions are gender equitable (Forsythe et al. 2016). Examining the gendered roles within social groups, the trait and variety preferences related to these roles, while documenting access to resources for cassava production and processing, are therefore important, especially from an equity perspective.

Nigerian cassava breeding programs prioritize “common denominator” traits in setting breeding agendas, designed to impact the largest possible number of people through improved varieties. Traits such as yield and resistance to pests and diseases have been prioritized over and above others. This “one size fits all” approach has been successful, with the proportion of improved varieties in farmers’ fields largely increasing over 1998-2009 (Oparinde et al. 2016; Wossen et al. 2017). Further increases in adoption rates can lie with responding to more nuanced, and contextual needs and associated traits, which have been hitherto largely low in breeding priority. Tensions between breeding paradigms that tend to reduce the number of traits targeted, and the wide array of traits emerging from diversity of uses and users is a challenge for breeding programs, which can be overcome in part by careful diagnosis of needs, goals and livelihood strategies of women and men involved in production and processing of the target crop (Christinck et al. 2017). Yet, gender specific crop trait preferences are seldom studied or prioritized in breeding programs (Asrat et al. 2010). If the preferences and needs of an under-served group are included in new varieties it can contribute to food security equity and empowerment of a leading food producing sector composed of many small businesses. Prior studies in farmer preferences in cassava varieties (Chiwona-Karltun et al. 2015) and gender-differentiated trait preferences in cassava showed linkages between traits and livelihood strategies (Chiwona-Karltun et al. 1998). Gender analysis of trait preferences in small scale Nigerian farmer/processor systems is relevant in this complex production/processing/consumption axis, as sex, religion, age, education, ethnicity, and marital status can all have an impact on how different men and women relate to the crop (Shields 2008).

The objective of this study is to diagnose gender-differentiated preferences around cassava traits and varieties, while examining gender based constraints in accessing and adopting cassava planting material. This study will inform gender responsive breeding strategies, specifically to help set breeding priorities and to expand the potential impact of improved varieties. We worked with eight communities in Nigeria engaged in joint small-scale farming and processing to identify: the varieties cultivated by men and women and the factors that influence these choices; traits preferred by men and women in the varieties that they grow; mechanisms for accessing cassava planting material, and challenges/opportunities related to these.

## Methodology

The study was carried within six villages in the Southwest, Pontela-Akinola and Elere Adeogun (Oyo state), Oba Oke and Agodo Owode (Osun state) and Ibooro and Agbetu (Ogun state), and two villages in the Southeast, Umuoso and Imerienwe, in Imo state. These villages were randomly selected based on a scoping study conducted to identify villages within each state with high rates of cassava production, processing activities/centers, and marketing outlets for fresh roots and products.

### Scoping Study

Prior to the main study, 29 villages were randomly selected from Osun (10), Ogun (7), Oyo (6) and Imo (6) states. Key informant interviews were held with extension officers. Focus group discussions were held with farmers and processors in villages in the selected states. Vital information elicited assisted in identifying key cassava producing, processing, and marketing areas within each state. Village leaders were contacted for community engagement and the mobilization of farmers and processors for focus group discussions and individual interviews.

### The Main Study

Quantitative semi-structured individual in depth interviews (IDIs) and focus group discussions (FGDs) were both used for data collection. All the respondents from the IDI’s in this study were either small scale farmers or farmer/processors. We define a farmer/processor as an individual who farms cassava, but is also involved in the processing into local food products. Data collection for the main study took place between August 2014 and January 2015. A total of 16 FGDs were conducted. In each community two FDGs were held: one with women and one with men. FGDs had 10 participants, with some minor deviations in some cases. A total of 150 in-depth semi-structured interviews were conducted with individual men and women to obtain quantitative information on household demographics, varieties cultivated, trait preferences, as well as access to and control of productive resources. Each respondent was asked which varieties he/she cultivated, then for each variety the respondent indicated what particular traits motivated them to cultivate that variety.

### Data Analysis

Similar patterns of responses from all interviews on preferred traits were coded into themes. Themes were based on the type and diversity of the traits provided, using a coding tree. Frequencies and percentages of the number of times a coded trait was mentioned were calculated. Stem sources were pre-coded into: “Own farm,” “gift from other farmers in same village,” “gift from other farmers in other village,” “buy from farmers in same village,” “buy from farmers in other village,” “research stations in the state,” “research stations outside the state,” “from extension/ ADP,” “from NGOs, projects, cassava multipliers.” Quantitative data on traits and stem source from the individual interviews were analyzed using Chi-square tests and in the few cases where more than 20% of the counts where less than five, a Fischer exact test was used to correct the Chi-square test. Significant differences between ratio variables were calculated using an independent t-test. FGD narratives relating to the rationale for preferred varieties were analyzed using context analysis, to complement findings from individual interviews.

## Results and Discussion

### Demographic Characteristics of Respondents

The respondents (for both IDIs and FGDs) had cassava-related agricultural (farming, processing) activities as their primary occupation as this was the criteria for selecting the participants, as well as a few civil servants who had farming as their secondary occupation. For women secondary occupations included trading, operating milling machines, tailoring and hair dressing. With the exception of a few single men and a few widows all respondents were married. The secondary occupations for the men included artisanship, livestock rearing, hunting, butchery, and commercial motorbike riding business. From the FGDs, it was clear that each community cultivated different fruit, tree, cash, food, and vegetable crops, but cassava cultivation formed the bulk of the food products produced across all communities.

**Fig. 1:**
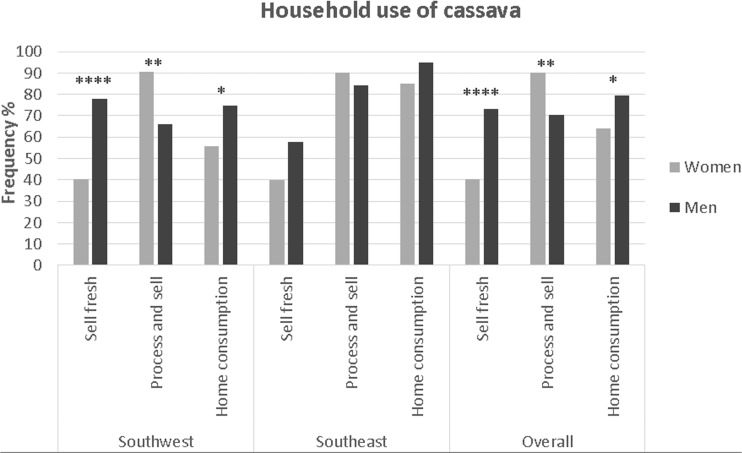
Household use of cassava.

Analyzing all study sites together, significantly more women processed and sold cassava products then men, while men mentioned sale of fresh roots significantly more often (Fig. 1). Closer examination of trends in the Southwest and the Southeast separately revealed this effect to be more pronounced in the Southwest (Fig. 1). There was no evidence of differences between women and men for processing involvement in the Southeast. Processing activities in the Southeast are more integrated with the whole household usually involved in home level processing. This situation is reversed in the Southwest, where processing centers outside the home are common, and cassava producers go to these centers to process their roots for a fee (Abdoulaye et al. 2015). In West Africa, especially in Nigeria, women dominate cassava processing and are largely responsible for marketing products (Walker et al. 2014). Yet, this gender role has different effects depending on the regions, markets, and resources available to women. It appears that when the processing centers are located outside the home (such as in the Southwest), the gendered roles in cassava production and processing are more pronounced.

### Varietal Diversity and Preferred Varieties

Using names provided by respondents, a total of 55 varieties were identified across the eight sites included in this study (Table 1). Interestingly, while 34 varieties were identified from six sites in the Southwest region, 23 varieties were identified from only two sites in the Southeast. Comparing data from the IDIs for the Southwest and the Southeast, farmers in the Southwest cultivate significantly more varieties per farmer (an average of 3.41 compared to 2.63, *p* < 0.001 independent t-test). This indicates a greater diversity and range of varieties grown in the Southeast, compared to the Southwest of Nigeria.

**Table 1:** THE 55 VARIETY NAMES ENCOUNTERED IN THIS STUDY. Varieties names 1 through 34 were mentioned in the six study sites in the Southwest, while varieties 35-55 were mentioned in two study sites in the Southeast. The name 419 + and Vitamin A ++ were both mentioned in the Southwest and the Southeast.

Southwest				Southeast			
1	419	18	Mokosokun	+	419	51	Nwaocha
2	Aboyade	19	Molekanga	35	Afdrimi	52	Nwaogomi
3	Adelowo	20	Odongbo	36	Afojiaku	53	Nwokoum
4	Agric	21	Ohori	37	Chigazu	54	Onye Ocha
5	Oko Iyawo	22	Okin	38	Dabiri	55	Umucass 38
6	Arubielu	23	Olosumeje	39	Imi Anwuru	++	Vitamin A
7	Atu	24	Omolewe	40	Imo Best		
8	Ayeke	25	Otegbeye	41	ISADAP		
9	Dangaria	26	Oyarugba	42	Ishiaghiama Oke		
10	Ege Dudu	27	Sajobo	43	Katikati		
11	Ege Funfun	28	Sanni	44	Mgboto Umuahia		
12	Greengate	29	Sunday	45	NR		
13	Ibikunle	30	Texaco	46	Nwa Adacho		
14	Idasa	31	Tokotaya	47	Nwageri		
15	Idileruwa	32	Tokunbo	48	Nwankwo		
16	IITA	33	Tomude	49	Nwanma		
17	Koforogun	34	Vitamin A	50	Nwanuhie		

The study sites in the Southeast were proximal to the National Roots Crops Research Institute in Umudike, where all officially registered new cassava varieties are available after release. These communities may therefore have greater opportunity to participate in on-farm trial evaluations and thus have access to new varieties as they are released (Oparinde et al. 2016). Larger socio-cultural factors could also be at play. Cassava production in Southeast Nigeria largely takes place on small plots of land owned by resource poor farmers. Korieh (2010) shows the size of farm holdings in Imo State (0.07 ha average) were below the national average of 0.57 ha. Land scarcity is driven by traditional lineages of communal land ownership, under which lineage heads allocate land each year to the households making each household more and more dependent on a smaller piece of land. Seeking actively for higher yielding varieties can therefore be an extra incentive under these constrained conditions.

Women in the Southeast are more empowered compared to women in other regions of Nigeria (Ayevbuomwan et al. 2016). This is reflected in cassava production; cassava in Imo state (Southeast) has been an important crop for women since the 1920’s as a food security and subsistence crop, but also as a means to generate income independently (Korieh 2010). Yams used to be the “king of crops” in the Southeast and have traditionally been associated with masculinity, reflected in typical male names such as Ezeji (Yam king) (Korieh 2007, 2010). Yams have been largely replaced by cassava production by women, and cassava is now referred to as the “mother of all crops.” Genetic diversity of cassava has been linked to marriage exchanges, where kinship structures influence seed exchanges and diversity of cassava in Gabon (Delêtre et al. 2011). The “feminization” of cassava farming in the Southeast, coupled with high levels of women’s empowerment and access to diverse sources of planting material through kinship structures may finally explain the larger diversity and range of cassava varieties grown in the Southeast.

### Ranking and Reasoning for Preferred Varieties

FGD participants were asked to rank the varieties grown in each location. Comparing the ranking of varieties between men and women FGDs revealed different patterns based on location (Table 2). These results show that farmers cultivated several varieties at a time and allocated their largest fields to their most preferred variety. There was mostly agreement between women and men in ranking preferred varieties within each location. Reasons for selecting and growing preferred varieties differed between men and women farmers (Table 3). In Pontela-Akinola, the reasons for women and men tended towards preferred traits such as high yielding and early maturity in Molenkanga:

**Table 2:** SEX-DISAGGREGATED RANKING OF THREE MOST PREFERRED CASSAVA VARIETIES ACROSS STUDY SITES.

	Ranking	1st	2nd	3rd
Location				
Southwest				
Pontela Akinola	*Men*	Molekanga	Oko iyawo	Arubielu/Egedudu
	*Women*	Molekanga	Odongbo	Oko iyawo
Elere Adeogun	*Men*	Dangaria	IITA	419
	*Women*	Dangaria	IITA	Odongbo
Agodo Owode	*Men*	Oko iyawo	Ege dudu	Adelowo
	*Women*	Idileruwa	Adelowo	Ege dudu
Agbetu	*Men*	Texaco	Onigidudu	Olusumeje
	*Women*	Lufodo	Idileruwa	Texaco
Oba Oke	*Men*	Arubielu	Oko iyawo	Ege funfun
	*Women*	Oko iyawo	Arubielu	Tomunde
Ibooro	*Men*	Idileruwa	Odongbo funfun	Dajofolowo
	*Women*	Idileruwa	Oko iyawo	Aporo-ofo
Southeast				
Umuoso	*Men*	Nwaocha	NR	Katikati
	*Women*	Nwaocha	NR	Katikati
Imerienwe	*Men*	Nwankwo	Chigazu	Nwaonuhie
	*Women*	Nwageri	Chigazu	Nwankwo

**Table 3:** SEX-DISAGGREGATED RATIONALE FOR CASSAVA VARIETY PREFERENCES. Listed reasons have been summarized from FGDs in all study sites.

Name of variety (type)	Reasons for preference Men	Reasons for preference Women
Molekanga	high yielding, poundable, good for *gari*, marketable, early maturing (6-9 months). Also called poverty removal crop	poundable, root size, high yielding, weed suppression, low cost of production and early maturing. Also called food security friendly cassava variety
Oko Iyawo	poundable, mealy, high yielding, early maturing (7-12 months) and resistant to pest and diseases	mealy, short time to cook, good taste and product quality for *gari, eba, fufu* and *lafun*
Dangaria	good taste, white color, very tall with multiple stems for planting materials. Good for feeding livestock	high market demand, poundable, good root and product color, weed suppression, tall stems, good product quality for *gari, fufu,* and *lafun*
Idileruwa	resistant to pests and diseases, in-ground storability without rotting, weed suppression, low cost of production	can survive after pest attack, underground storability without rotting, can stay for 3-4 days after harvesting, good product quality
Nwaocha	de-waters faster, high dry matter, late maturing, allows for intercropping	beautiful to behold, good plant architecture, ferments quickly 2-3 days, odorless, good product quality for *abacha, lafun* and *gari*
Nwankwo	high yielding, marketable and early maturing	good product quality, high root number and early maturing
IITA	pest and disease resistance, root size and shape, branches well and smothers weeds, can survive harsh conditions	high yielding, post-harvest in-ground storability, high dry matter content makes *gari* swell.

“*At 6-9 months the variety is good enough to harvest and it gives quick money at a profitable price. That is why it is called ‘Molekanga’ i.e., a lazy person can make furniture from the income.”*

Pontela-Akinola women FGD.

In Elere-Adeogun, Dangaria was ranked first by women; they explained that its young leaves were used to prepare soup/sauce. While women focused on the taste and early maturing traits, men preferred high yields because they use Dangari for livestock feeding (Table 3). Men and women both preferred its cooking quality traits. A similar result was seen for the variety Nwaocha in Umuoso. While men mentioned the product quality, the description by women of the quality attributes for products was more informative:

*“Nwaocha is good for food products such as* abacha, akpu *and* gari *due to its fine white color. Unlike* gari *and* akpu *that may sometimes have dull white color,* abacha *must have bright white color which can only be got from Nwaocha variety.”*

Umuoso women FGD.

The rationale for preferences of the variety IITA was also interesting. Men preferred IITA for its in-ground storability and high yield. This was different for women, who largely focused on early maturity, taste, and dry matter content (Table 3). While men and women did mention similar traits in varieties, the way these traits were expressed was often different. For example, while men just noted that they liked the variety that suppressed weeds, women went on to explain how it reduced the labor required for weeding:

“*It [Idileruwa] suppresses weeds because of the canopy. It helps in reducing weeding cost*” Agbetu women FGD.

Results from FGDs in Table 3 show that even though women and men often ranked the same variety similarly, their reasons for doing so were different. Women more often expressed product quality for *gari, eba, fufu* and *lafun* as a criterion, while men more often mentioned agronomic traits. This is in agreement with trait rankings from the IDIs (see below), and indicate a higher priority women place on product quality traits, reflecting the different roles men and women play in production and processing. Furthermore, the descriptions women gave to explain their preferences were often richer and more informative than men. The gendered division of labor in cassava production in Nigeria (Curran et al. 2009; Walker et al. 2014) likely equips women with superior tacit knowledge of cassava production and processing, akin to the folk taxonomic knowledge attributed to women as keepers of crop biodiversity (Howard 2003), including for cassava (Boster 1985).

### Cassava Traits

### Gender Specific Traits and their Intersection with Region

Unsurprisingly, cassava farmers across all study sites attached substantial weight to traits such as high yield, root size, early maturity, and dry matter content (Table 4). The yield of storage roots constitutes an important basis for farmers to cultivate the various varieties identified in the study sites. This finding supports the assertion that high yield is one of the primary traits in farmers’ varietal selection (Abdoulaye et al. 2013). There were however significant differences in the extent that classes of traits where mentioned by women and men (Table 4) across all study sites. Women attached greater importance to cooking/processing traits then men (*P* = 0.039). Statements coded in this category included: “*Makes good products gari, fufu and lafun;” “Products made from it swell and draw and mold fine;” “It ‘fills’ the stomach when eaten and fufu and gari made from it draws;” “Gari made from it is appealing.”* These statements closely follow and validate the FGD data for reasons of preference when ranking varieties (Table 3). Conversely, men attached greater importance to agronomic traits than women (*P* = 0.033). Statements coded in this category included: “*Suppresses weeds;” “Good canopy formation;” “Beautiful, appealing in the field.”* Together these findings validate the assumptions that gendered divisions of labor in cassava production and processing directly drive trait preferences and accumulated knowledge, as described above.

**Table 4:** OVERALL CASSAVA TRAIT FREQUENCIES. Frequencies with which cassava traits are mentioned by cassava farmers in the study sites in the Southwest and Southeast of Nigeria. Traits are listed according to the frequency (from high to low) with which they were mentioned across regions. Data are then dis-aggregated by sex. For the dissagregation into regions only the traits that show significant differences between men and women are shown. Differences in frequencies between the sexes are tested for using Chi-square test.

Traits	Frequencies (%)			*P*-value
Overall	All respondents	Women	Men	
high yield	73.3	72.2	74.4	ns
root size	60.0	68.1	52.6	0.053
early maturing	55.3	54.2	56.4	ns
dry matter content/swells	42.7	43.1	42.3	ns
cooking / processing quality **+**	40.0	48.6	32.1	0.039*
flesh color	38.0	31.9	43.6	ns
post harvest shelf life	38.0	38.9	37.2	ns
poundability	37.3	34.7	39.7	ns
fast cooking	32.0	29.2	34.6	ns
good price/marketability	29.3	26.4	32.1	ns
agronomic characteristics++	26.0	18.1	33.3	0.033*
taste	24.7	30.6	19.2	ns
resistance to pest and diseases	20.7	18.1	23.1	ns
adaptation to extreme weather condition	10.0	9.7	10.3	ns
labor requirement	4.7	5.6	3.8	ns
*Southwest*				
flesh color	30	19	39	0.023*
good price/marketability	22	14	28	0.050
agronomic characteristic++	20	8	30	0.003**
*Southeast*				
Fast cooking	13	25	0	0.047*

^**^p value<0.01, ^*^p-value<0.05, ns = not significant

**+**This group represents: “Easiness and suitability to make the food products gari, fufu and lafun” (64%) and “Products made from it swell and draw and mold fine” (18%). Others are “It ‘fills’ the stomach when eaten and fufu and gari made from it draws” (6%), “Gari made from it is appealing” (6%) “Roots after processing are not watery” (3%), “It is easy to process” (1%), “It is soft when boiled” (1%), “It does not have a smell when fermented (1%),

**++**This group represents “weed suppression and good canopy formation” (80%,) others are “beautiful, appealing in the field” (16%), “likes waterish areas” (2%), “stems can be stored for long” (2%)

There were interesting differentiations between men and women’s preferences within regions. In the Southwest, flesh color and agronomic characteristics had significantly higher frequency amongst men (*P* = 0.023 and *P* = 0.003 respectively). This could directly reflect the high significant proportion of fresh sale of cassava roots by men (Fig. 1), who would therefore pay greater attention to fresh market traits such as flesh color. In the Southeast, fast cooking had higher incidence amongst women than men (*P* = 0.047). This could be related to the overall higher rate of home consumption in the Southeast (Fig. 1), together with the importance of cassava products typical of the Southeast such as *abacha*, that involves cooking (Etejere and Bhat 1985; Iwuoha et al. 1996). *Abacha* is grated fermented thin cassava pieces often consumed as snack or made into a salad.

Variations in time to maturity played an important role in the farming systems of cassava farmers across study sites and therefore constituted one of the major traits for which farmers cultivated varieties. FGD results indicated that even though late maturing varieties store longer in the soil and serve as collateral or savings for income in times of necessity, earlier maturing ones were preferred among all respondents across sites as a source of quick food and income. The rationale for early maturity for women was linked to the underlying food security for the household in mind:

“*Few varieties have been abandoned because they don’t mature fast, taking about three years to mature before harvest. We cannot wait for that long before we feed our children*”

Pontela women FGD.

The role of women as processors and sellers of cassava products can be considered leading in relation to their trait preferences for cooking/processing quality aspects. However even though men were much less involved in processing activities they also give high priority to these, illustrating that men were also well informed about the importance of processing traits as almost all the fresh cassava was bought to be processed. Both men and women farmers also considered profitable prices/marketability as the same and beneficial. This finding is similar to Asrat et al*.* (2010) who found that the ability of a variety to fetch a good price was an incentive to farmers’ selection of that variety. The CMS reports (Wossen et al. 2017) showed that one of the most important traits mentioned, especially by women, was “ease to peel” a task mainly performed by women. The fact that this was not mentioned as a trait in this study was probably related to the way variety trait preferences were assessed in the study areas. Instead of asking for preferred traits directly, this study asked farmers which varieties they grew, and asked which traits they liked most within that particular variety. This could be indicative that ease of peeling might not only be variety independent but more related to the season when harvested and to the age of the root. However, the large importance given to root size (Tables 4 and 5) can be related to the peeling work. Larger roots have a smaller surface to root weight ratio and therefore demand less peeling work.

**Table 5:** REGION SPECIFIC CASSAVA TRAIT FREQUENCIES. Frequencies with which cassava traits are mentioned by cassava farmers in the study villages in the Southwest and Southeast of Nigeria. Traits are listed according to the frequency (from high to low) with which they were mentioned by each sex. Data are then dis-aggregated by region. Differences in frequencies between the regions are tested for using Chi-square test.

Traits/gender	Frequencies %		P-value
Men and women	SouthWest	SouthEast	
high yield	67.6	89.7	0.0071^**^
root size	57.7	66.7	ns
early maturing	49.5	71.8	0.016^*^
post harvest shelf life	41.4	28.2	ns
cooking / processing quality	39.6	41.0	ns
fast cooking	38.7	12.8	0.0028^**^
poundability	35.1	43.6	ns
dry matter content/swells	33.3	69.2	0.000097^***^
flesh color	29.7	61.5	0.00043^***^
good price/marketability	21.6	51.3	0.00047^***^
taste	20.7	35.9	0.059
agronomic characteristic	19.8	43.6	0.0036^**^
resistance to pest and diseases	18.0	28.2	ns
adaptation to extreme weather condition	8.1	15.4	ns
labor requirement	4.5	5.1	ns
*Women*	*SW*	*SE*	
root size	67	70	ns
high yield	67	85	ns
early maturing	52	60	ns
cooking / processing quality	48	50	ns
post harvest shelf life	38	40	ns
dry matter content/swells	31	75	0.00069^***^
fast cooking	31	25	ns
poundability	27	55	0.025^*^
taste	25	45	ns
flesh color	19	65	0.00019^***^
resistance to pest and diseases	13	30	ns
good price/marketability	13	60	0.000060^***^
adaptation to extreme weather condition	10	10	ns
other agronomic characteristic	8	45	0.00070^***^
labor requirement	4	10	ns
*Men*	*SW*	*SE*	
high yield	68	95	0.019^*^
root size	49	63	ns
early maturing	47	84	0.0050^**^
fast cooking	46	0	0.00027^***^
post harvest shelf life	44	16	0.027^*^
poundability	42	32	ns
flesh color	39	58	ns
dry matter content/swells	36	63	0.034^*^
other cooking / processing quality	32	32	ns
other agronomic characteristic	31	42	ns
good price/marketability	29	42	ns
resistance to pest and diseases	22	26	ns
taste	17	26	ns
adaptation to extreme weather condition	7	21	0.075
labor requirement	5	0	ns

^***^p value<0.001, ^**^p value<0.01, ^*^p-value<0.05, ns = not significant

#### Regional Preferred Traits and Intersections with Gender

When comparing trait differences between regions (Table 5) there were significant differences in the manner two major traits were valued: respondents in the Southeast attributed more value to “high yield” and “early maturing”. Both can be understood by the limited available land and the relatively smaller plots in the Southeast (Korieh 2010) necessitating preference for better value in unit space and time. When land is scarce early maturing varieties facilitate more harvests per time unit and optimizing yield is the only possibility to increase production. The scoping study reveals that this is also reflected socially in the presence of specific harvesting/processing/marketing days in certain parts of Imo and Abia state related to cassava creating a social ‘effervescence’ (Collins 2004) to get heavy work done effectively. Persons harvesting outside of such days are sanctioned.

Fast cooking is mentioned more in the Southwest than the Southeast. This might be ascribed to the greater role of boiled, pounded, or roasted cassava in the Southwest. Proximity to the international border between Nigeria and Benin hosts a substantial amount of immigrant farmers and farm laborers from Togo and Benin who play a substantial role in sustaining the agricultural sector through direct production and as source of farm labor (Agbonlahor and Enilolobo 2013). In Benin and Togo freshly boiled and pounded cassava is common (Nago and Hounhouigan 1998). Some varieties, like Atu, were sometimes also referred to as ‘Cotonou’ and are suitable to boil/roast and to pound. Only two of the respondents for the quantitative individual interviews in our study were from Togo. The influence of the immigrants from Benin and Togo might have made boil, pound, roast, or even eat fresh in the field as a snack or even as a meal at home more popular in the Southwest.

Table 5 shows that “dry matter content/swells,” “flesh color,” “good price/marketability,” and “agronomic characteristics” were more frequently mentioned in the Southeast. This lays credence to the larger role that cassava takes up within the livelihoods of farmers and farmer/processors in the Southeast. The more frequent mention of agronomic characteristics is comprehensible in a setting where small scale farmers and especially women are involved in cassava cultivation. This reasoning is strengthened as the specific role of women in cassava production and processing in the Southeast is illustrated when we consider only women (Table 5): “dry matter content/swells,” “flesh color,” “good price/marketability,” and “agronomic characteristics” almost all become even more significant or at least do not lose in significance. When only considering men, we see that the general observed trend when considering men and women together is maintained, but that the trait “post-harvest shelf-life” is significantly more important for men from the Southwest. This can be explained by the larger markets and larger-scale production present in the Southwest involving transport over longer distances in which usually more men than women are involved.

### Access to Stem Sources and Decision Making around Planting Material

#### Stem Sources

**Fig. 2:**
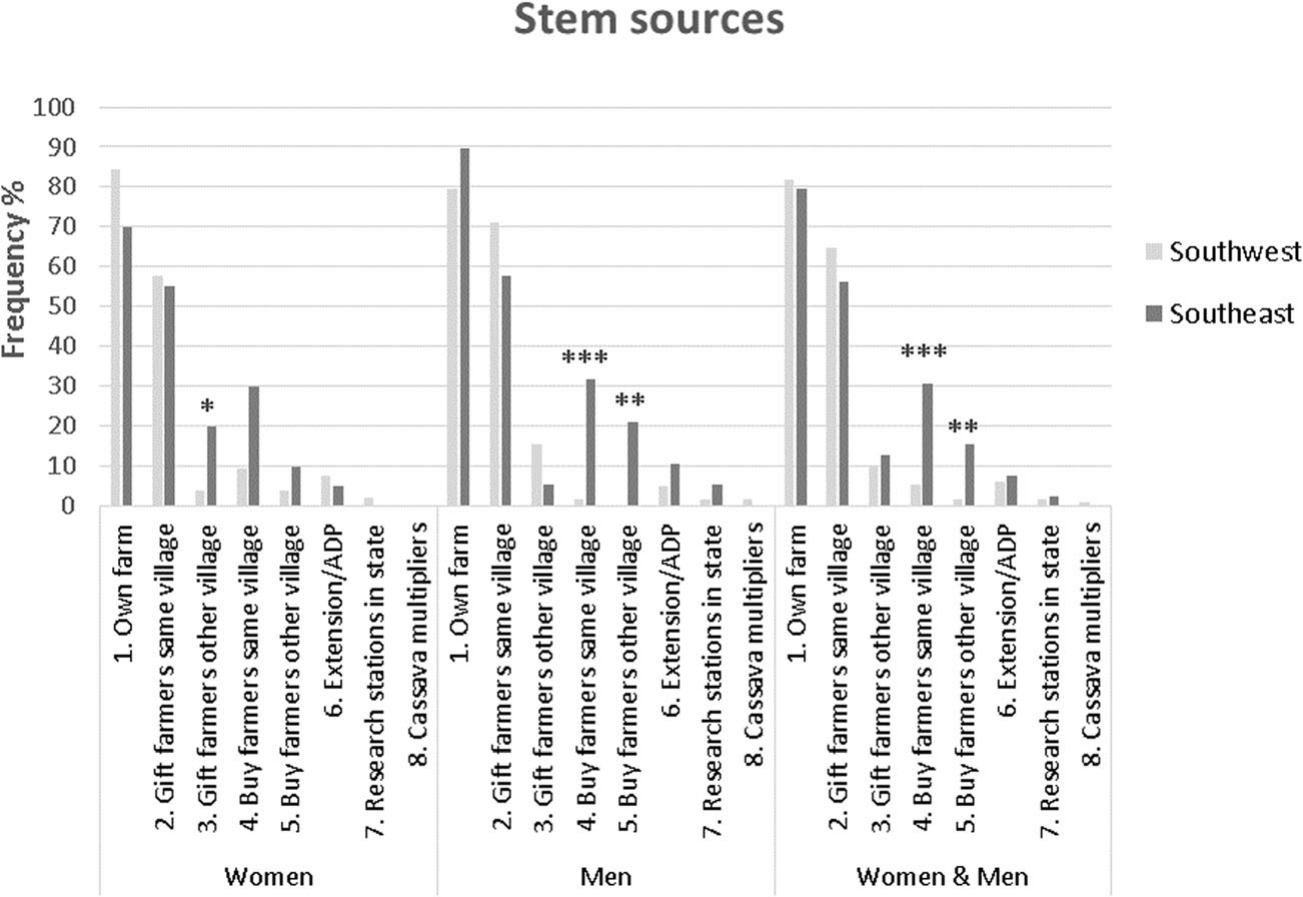
Frequencies of cassava stem sources.

The main stem source for men and women farmers was their own farm and neighbors through gift or purchase (Fig. 2). A similar study in Uganda on cassava farmers’ source of seeds indicated that about 89% obtain seeds from informal sources, for the most part from their own saved seeds, the local market, and neighbors (ISSD Uganda 2014). This reliance on informal seed systems held in an analysis of five countries across sub-Saharan Africa (Mcguire and Sperling 2016). Figure 2 shows that stems were mostly obtained from “own farm” and as a gift from other people in the village. Within these two categories there were no significant differences when comparing regions for both sexes together**.** However, significant differences appear for the sources “Buy from farmers in the same village” and “Buy from farmers in another village” that were both mentioned more often in the Southeast. This is hard to explain other than that plant material is scarce as all plots are intensively cultivated while in the Southwest extensive cultivation for stems is possible as land is less scarce. When only considering men, the same pattern appears which is understandable as men will not be much involved in rotating working groups that provided labor for each of the members’ plots without any money or in-kind transaction. In the Southeast, women form such work groups along the lines of kinship, age grades, friendship circles, or social or finance clubs, and provide an important means of labor for cassava production (Korieh 2010). These work groups could provide a conduit of exchange for planting materials, and among such women groups free exchange of stem material would be more common than among men.

#### Intersection with Religion

Keeping intersectionality in mind, we disaggregated stem sources by religion. Respondents in the Southeast were all Christian while respondents in Southwest included both Muslim and Christian. For the stem source within the Southwest, Muslim women mentioned “Gift from another farmer in the same village” less often than Christian women. Furthermore, Muslim men mention “Gift from other farmers in the same village” more often than Christian men (Table 6). When only considering Muslim respondents, a very significant difference appears between Muslim women and men: Muslim men mentioned “Gift from other farmer in the village” much more than Muslim women. Muslim men also mention “Buy from other farmers in other village” far less than Christian men. This suggests that Muslim men may maintain very strong reciprocal relations while this is not as true for Muslim women, who often buy stem material. Adekunle et al. (2016) indicates the role of religion as having a possible large influence in the adoption and success of innovative technologies especially for cassava that is on the rise in popularity and significance. Taking note of religious norms and roles in relation to technology development and seed dissemination can therefore be important from an equity perspective as religion often sets specific norms and rules. These results are interesting, but difficult to explain with the current data set, and require significant further study.

**Table 6:** RELATIONSHIP BETWEEN SEED SOURCE AND RELIGION. Frequencies (%) investigating the intersection between seed source, gender and religion in the Southwest. The southeast had only Christian respondents and was therefore not included in this Table. P-values relate to Chi square test.

		Gift from other farmers in the same village		Buy from others farmers in same village		Buy from other farmers in other village	
		Freq.	*P*-value	Freq.	P-value	Freq.	P-value
*Sex*	*Religion*						
Women	Christian	66	0.019*	14	ns	4	ns
	Muslim	36		18		9	
Men	Christian	57	0.017*	16	0.017*	23	0.039*
	Muslim	82		0		0	
*Religion*	*Sex*						
Muslim	Female	36	0.00045***	18	0.020*	9	ns
	Male	82		0		0	

^***^p value<0.001, ^**^p value<0.01, ^*^p-value<0.05, ns = not significant

#### Decision Making around Planting Materials

There was a greater agreement on variety ranking in Umuoso and Imerienwe in the Southeastern region, than Pontela-Akinola and Elere-Adeogun in the Southwest between women and men (Table 2). A general norm for married women to consult their husbands before deciding on planting a new variety may inform women in the Southwest and may influence their decision to adopt and utilize it for cassava products:

“*If there is something new that is being introduced, we may not be quick to do those things but if our men do them, then we too will do them*”.

Pontela women FGD.

“*Men mostly decide on where to plant which variety because they gave portions of their lands to women to plant as well as the varieties to plant. Men also decide when to plant because it is unsafe for women to go to farm alone. Men have access to new varieties. If they are old varieties, the women take their own decisions on which one to plant because they source from the previous harvest*”

Adogo women FGD

However, this norm is less pronounced in the Southeast:

“*We don’t consult anybody before planting anything. Men don’t know anything about the cassava we are planting*”

Imerienwe Women FGD

This tendency for more autonomy in production, and decision making around planting material reflects the higher levels of empowerment experienced by women in the Southeast (Ayevbuomwan et al. 2016), especially around cassava production. These “cassava queens” (Korieh 2007) hold an important place in the social fabric of the communities, and may experiment and introduce new varieties. Our results concerning greater varietal diversity in the Southeast can also be linked to this increased decision-making power of women around planting materials.

## Conclusion

This study appears at an opportune moment in cassava breeding in Africa: unprecedented investment in cassava research, coupled with greater donor interest and insistence on gender responsive agricultural research (BMGF 2012). By filling a niche in current knowledge around cassava varietal and trait preferences in two key cassava producing regions in Nigeria, we provide information to help breeders answer the question: what other traits can we breed for? With the application of latest technology advances to cassava, such as genomic selection (Wolfe et al. 2017) and high throughput processing technologies (Ikeogu et al. 2017) cassava breeding stands at the dawn of major advances and greatly accelerated breeding cycles, leading to more rapid development of improved varieties.

It is at this juncture that setting the “next generation” of breeding priorities is critical. New breeding methods offer the opportunity to expand the range of traits prioritized. Technologies such as genomic selection, increasingly applied to cassava breeding, allow for a greater number of traits to be included in selection indices (Jia and Jannink 2012). Combined with advances in cassava product quality phenotyping (Sanchez et al. 2014), traits such as “cooking/processing quality” that are highlighted by this study become realistic targets for cassava breeders. This could lead to new breeding populations that introgress cooking/processing quality onto high yielding, early maturing backgrounds. Currently cooking/processing traits are low in breeding priority but by highlighting their importance, especially for women, this study builds a case for specific breeding targets, and incentivizes breeding programs to invest resources in these around this.

Lastly this study reveals interesting findings on differential varietal and trait preferences between men and women, as well as impacts of cross-sectional factors such as region and religion on traits and stem source. Given existing evidence of a large task division between men and women within cassava production and processing, differential trait preferences could be expected as evidenced by this study. Breeding programs can use this evidence to meet donor, national, and regional requirements for gender responsiveness, by targeting traits such as cooking/processing quality. Furthermore, differences in access to stem source amongst men and women, and the impact of religion on this process, can help inform targeted dissemination strategies and delivery packages. Ensuring equitable access to new products of cassava breeding programs would therefore include a two-pronged strategy of incorporating traits important for women, together with identifying entry points into local seed systems to ensure the new material reaches the fields of those who need it the most.
